# The Hospital Nursing Supplement

**Published:** 1894-07-21

**Authors:** 


### "The Hospital" Nursing Mirror.

Being the Extra Nursing Supplement of "The Hospital" Newspaper.

[Contributions for this Supplement should be addressed to the Editor, The Hospital, 428, Strand, London, W.C., and should have the word "Nursing" plainly written in left-hand top corner of the envelope.]

### News from the Nursing World.

### WHERE SHOULD THE FLOWERS GO?

"I have given up sending flowers now, because I have been told that at some of the children's hospitals the nurses are quite overdone with them," said a kind-hearted lady somewhat illogically. Because there are too many floral offerings made to one small pet institution, it is certainly hard that all others should be deprived of beauty. It saves trouble to send things to the nearest hospital, or just to drive with them to one in a respectable neighbourhood, but flowers are naturally more highly valued in ugly, dreary districts where visitors are few and gifts still scarcer. There are many workhouses and infirmaries where hampers of flowers and fruit are unknown luxuries, and certainly plenty of district nurses would gladly receive for the sick poor whom they tend in their own homes any fresh flowers, fruit, or vegetables—almost unknown luxuries to many of these invalids. The window-boxes at the West-end, so lovely in July, become a dreary spectacle of neglect on succeeding months when "the family" is superseded by caretakers, quite indifferent to the claims of the flowers for ordinary care and attention. If only some of the plants were sent to gladden the hearts of the sick poor they would give joy altogether out of proportion to the small amount of trouble which the kind act might entail on the donor.

### WORKHOUSE INSPECTORS.

That the time has come for women to be appointed as inspectors of workhouses seems to be acknowledged by many who speak with the authority of experience in such matters. If this wise suggestion be carried out, it is most important that the right class of woman should be chosen, and this can only be secured by careful selection and by adequate remuneration. The matron, or assistant matron, who is a fully qualified nurse, with a thorough knowledge of sanitation and sanitary law, would, of course, be competent to fulfil all requirements. Such a woman is not easily secured, yet no less competent a person ought to be accepted; for the holder of half-a-dozen easily-gained certificates for short courses of "training," whether theoretical or practical, lacks the knowledge of even the elements of intelligent inspection. Practical acquaintance with technical items, cultivated powers of observation, and above all, *tact*, that indescribable gift which is indispensable to successful administration, are all needed in the trained nurse in whom we look forward to finding the ideal inspector.

### MASSAGE.

We are often consulted on the subject of Massage, by would-be learners who demand to be told the cheapest way to secure the necessary certificate for practising and teaching this art, and we are interested to find the answer frequently given by us is entirely in sympathy with the authoritative utterance of the *British Medical Journal* of 14th inst.—"the legitimate massage market is overstocked, and that no woman, unless she has a private connection, has the slightest chance of getting a living by massage alone—at all events in London." This sentence occurs in a short article on "Immoral 'Massage' Establishments," which should certainly be perused by all who desire to take up medical rubbing as a means of livelihood that they may be warned in time wherein the dangers lie.

### CONVALESCENT HOME AT OXTED.

Mr. Passmore Edwards last week laid the foundation stone of a convalescent home which he is having built for the benefit of patients from Charing Cross Hospital. A charming site has been secured near Oxted, which is situated in a lovely part of Surrey, and a convenient and commodious Home is now in course of erection. It is to contain twenty beds for men, the same number for women, and ten cots for children. There will be an extensive view of the surrounding country from the new Home, which will stand on land recently purchased by Mr. Passmore Edwards, and known as the Limpsfield Chart farm. The plans include recreation-rooms, wards, offices, and also "quiet rooms" for men and for women—a considerate provision on the part of the founder.

### DISTRICT NURSING AT CAMBERWELL.

The last annual report of the Camberwell District Nursing Association is a particularly interesting one, and the increase in the work done by it is in due proportion to the enlargement of its premises. The association is affiliated with the Queen's Jubilee Institute, and has been established for the last four years, "and the full meaning of what is implied in the term 'skilled nursing' becomes yearly more understood." The report proceeds briefly to explain with admirable clearness that the work of a true nurse is not confined to nursing, her opportunities of influence and teaching being exceptional ones, "the position of the nurse constitutes her a most forcible teacher of hygiene . . . and often when her actual work is done, there lives on in the household she has ministered to abiding evidence of the value of her example and teaching." There is a Samaritan Fund in connection with the home at Camberwell, which enables many patients to enjoy a convalescent change and much needed rest before returning to their lives of toil, and the advantages accruing from this arrangement are well-nigh incalculable, both from the medical and nursing point of view. The importance of the work done at Dulwich by the Queen's nurse from Camberwell was thoroughly recognised by the whole community, and East Dulwich has now its own Queen's nurse, and contemplates securing the services of a second one.

### NURSES AT NORTHAMPTON.

The seventeenth annual report of the Northamptonshire Institution for Trained Nurses, affiliated with the Queen's Jubilee Institute, has been recently issued. The Lady Superintendent, Miss Wathen, is assisted by a staff of sixteen private nurses and five probationers, besides two Queen's nurses, and she is responsible for the invalid loan department. The committee have decided to give two years' training to all their probationers instead of the one year formerly granted, and three probationers are now at Edinburgh Royal Infirmary, whilst another is in training at Worcester Infirmary. The report says that the private nurses have been well employed during the year and much appreciated by their patients, and the Queen's nurses have done excellent work amongst the sick poor in their own homes. The committee express unqualified approval of their Superintendent, and congratulate themselves on securing her services.

### INCREASED SALARIES.

At the weekly meeting of the Board of Guardians at Hastings it was proposed and unanimously agreed to that the salary of the matron should be raised from £40 to £45 a-year, the Chairman remarking that he had never known of any kind of complaint being made against the matron; and he considered the Board had great cause to congratulate themselves on possessing so efficient an officer.—The Tavistock Guardians have raised the salary of their nurse from £20 to £23 per annum, the medical officer remarking that she was very careful, kind, and efficient.

### WOMEN GUARDIANS FOR NEWTON ABBOTT.

An interesting meeting was held in the Royal Public Hall at Torquay on the 12th inst., when Dr. Ley made a spirited speech respecting the condition of Newton Abbott Workhouse. His remarks were received with much cordiality, and at the close of the meeting it was proposed by Mr. J. E. Moon, and seconded by Mr. W. Pridham, "That this meeting is of opinion that it is most important that the ratepayers of the six wards shall elect in November next only those as guardians, of whom two at least shall be women ratepayers, who will pledge themselves to vote for more humane treatment, proper classification of inmates, separation of the sick under a sufficient number of thoroughly qualified officials."

### WORKHOUSE ATTENDANTS.

On July 12th a conference of the Workhouse Attendants' Association was held, under the presidency of the Countess of Meath, who explained the objects of the association. The Hon. Secretary said that eleven probationers had been accepted since the formation of the society last October. In addition to undertaking the care of the aged and infirm, these attendants will be available for the service of epileptics, imbeciles, and infants.

### A TRAINED NURSE FOR CORWEN.

The great success which has followed the introduction of Queen's district nurses at Bangor has encouraged some of the residents of Corwen to consider the question of securing a trained nurse for their own town. Dr. Price, of Bangor, attended a public meeting held recently, in the Assembly Rooms, and made an excellent speech, demonstrating the necessity for a fully-trained and qualified nurse being placed in every district. Mr. E. C. V. Lloyd, of Rhagatt, was in the chair, and endorsed the remarks of Dr. Price. A resolution was proposed by Mr. D. Lloyd, and seconded by Mr. C. P. Purchase, "pledging the meeting to support the movement."

### THE BUDAPEST CONFERENCE.

The Tropical Section of the International Congress of Hygiene and Demography, which is to be held at Budapest, September 1st to 9th, has paid Miss Nightingale the compliment of electing her as an honorary president. Already a large number of papers have been promised, and the section is likely to be the scene of some important discussions on the spread of cholera, results of opium consumption, and tropical colonisation. All information regarding the arrangements for the Tropical Section can be obtained from the Hon. Secretary, 40, Curzon Street, London.

### COSTLY BEEF TEA.

"I always make my beef-tea in the oven. I put it in an earthenware jar, and let it do slowly, as my mother and grandmother did," says a conservative, whose wholesome beef-tea won a doctor's commendation. But there are other ways of cooking it, both over and beside the stove, which, doubtless, ensure equally happy results. It is, however, in a provincial report that we learn for the first time that 14 lbs. of beef a week is the amount of "relief" needed for an old woman of 89. It would be worth while to learn from what cookery book she or her friends cull their recipe. The meat must of course be converted into essence, for no one could believe that any woman in her ninetieth year would be able to dispose of 2 lbs. of beef per diem in a solid form. Guardians who allow the sick inmates of their workhouse to be inadequately nursed, yet apparently consider, as recorded in the press, that an extraordinary amount of nourishment is necessary for recipients of outdoor relief in some cases at any rate.

### A MEMORIAL HOSPITAL.

The General Assembly, in appealing to the members of the Church of Scotland for a collection on a recent Sunday, specially mentioned the Lady Grisell Baillie Memorial Hospital. It has been so named in memory of the Church of Scotland's first deaconess, "so that deaconesses there trained may combine skill and experience in sick nursing with such spirit and service as that of her whom the hospital commemorates." It is suggested that others besides those intending to be deaconesses may be glad to avail themselves of the training procurable at the Memorial Hospital, for "to know how to nurse is now the ambition of almost all who visit the sick."

### SHORT ITEMS.

It is reported that a nurse has died of typhus in the Bicêtre Hospital.—The Rev. I. Conway Walter, of Langton Rectory, is appealing for funds for the Woodhall Spa Home for Gentlewomen, the office of which is at 32, Shaftesbury Avenue, London.—Mrs. Robb, *née* Isabel A. Hampton, graduated at the BelleVue Hospital, New York, and was Superintendent of Nursing at Cook's Hospital, Chicago, before she undertook the organization of the famous nurse training school at Johns Hopkins Hospital.—Miss Ada Regan has been appointed assistant nurse at Hastings Workhouse.—Miss Watson has been placed on the nursing staff of the Christchurch Workhouse, at a commencing salary of £28 per annum.—An excellent lecture and practical demonstration was recently given at Penrith by Nurse Rudd to the members of the Sick Nursing Association.

### On General Nursing.

By Rowland Humphreys, M.R.C.S., L.R.C.P.Lond.

XXI.—PERITONITIS (*continued*).

Applications to the abdomen at times relieve the pain of peritonitis. They may be either in the form of heat or of cold, or of a sedative, or even leeching in some cases. Some patients are unable to bear the least pressure, even the weight of the bedclothes causes pain. Yet in most cases poulticing, or fomentations, or the continuous application of a Leiter's coil with a current of water passing through it, heated to the maximum the patient can bear, and kept on for twenty-four hours at a stretch relieve greatly.

Ice may be applied in the form of a large iron cradle with pails of ice suspended from the bar. It is placed over the patient, and a blanket put over it; or an ice bag, or a poultice of pounded ice, or a Leiter's coil may be used. It is well, however, not to lower the patient's vitality too much by the use of cooling applications, as in all cases of peritonitis there is a tendency to depression of the vital functions.

Laudanum or other soothing applications may be placed on lint and applied to the abdomen.

The knees should be drawn up, where such a position is assumed by the patient, and supported in this position; and, if he desires to keep his arms out of bed above his head for the purpose of assisting his respiration by bringing into play the auxiliary muscles, he should have them covered by some suitable woollen material.

2. "Cases which may require operation."

Any case of peritonitis may be benefited by operation, provided that it is seen sufficiently early, and that the general condition of the patient is such that an operation is at all justifiable. The question always arises, sooner or later, will this case get well of itself, or must an operation be done? The time when it becomes necessary to perform any operation depends on the cause of the disease, on the condition of the patient, whether it be on the mend or the reverse, on the amount of adhesions it is desired should be formed before opening the abdomen, and on other things. So that it must vary widely. There are, however, certain cases which may at any time require operation. Amongst these are cases of obstruction to the bowels, of perityphilitis, of localised abscess which is in danger of rupturing into the abdominal cavity, of perforation or inflammation of a local nature in which adhesions are forming, and such like. These cases give an immense amount of anxiety, and require the most watchful care, so that any sign of approaching extravasation or rupture into the abdominal cavity may be noted, and an operation at once performed. Absolute rest is the great indication. This implies an absence of movement of both viscera and muscles of the body. The most anxious cases are, perhaps, those in which an operation has been performed, and in which any movement may start fresh complications, such as hæmorrhage.

3. "Cases in which an operation is inevitable."

These include most cases of internal strangulation as by a band, cases in which an abscess has ruptured into the abdominal cavity, or where a viscus has burst, as the gall bladder, or where one has been perforated, as by a stab, or where the abdominal cavity has been accidentally opened, and of course some septic material accidentally introduced into it.

In such cases as these the abdomen has to be opened, the rent in the viscus closed, or the abscess cavity evacuated, and antisepticised, and, if necessary, drained, and the abdominal cavity cleaned out. After an operation it is of first importance to avoid any degree of distension of the intestines, and if such should supervene at once to empty the bowel either by enema or by purgative. It is necessary that the bowels should be thoroughly emptied previous to any abdominal operation, in order to prevent, as far as possible, this dangerous complication.

The treatment of peritonitis is undergoing a steady improvement in respect to results. The operative treatment which has of late years come so largely into vogue, in now so successful and, in many cases, of so simple a nature that abdominal section, once the most fatal of operations, has become one of the safest. In some forms of the complaint, notably in tubercular peritonitis, the alteration in the general condition effected by simply opening the abdomen, and, perhaps, flushing it out, is perfectly marvellous, considering the intractable nature of this complaint if treated in any other way.

### Ambulance Work in Paris.

The interest attaching to a comparison of the various methods of rendering first aid to the wounded in use in different places is of a thoroughly practical character; and a periodical comparing of notes on the advantages and disadvantages of the several means employed could only lead to an all-round strengthening of weak points. The average Englishman is, perhaps, a little too apt to fancy that in all arrangements for the health of the community his country is far ahead of any other; but with regard to ambulance work, this comfortable belief is, unfortunately, ill-justified. It is Vienna, not London, that leads the way in this respect, much as has been done by St. John Ambulance Society and by the Hospitals Association—the last-mentioned association's service being supported by the generosity of one private individual. Neither, if one can judge by the number of inventions and improvements lately submitted to and approved by the Parisian authorities, is there any lack of interest in the matter in the French capital.

Among the above-mentioned inventions is a light ambulance, of which Dr. Barnay is the originator, supplied with three joints or hinges, "in such a manner as to allow it to turn even at right angles upon itself." The advantages claimed for this arrangement are twofold: Firstly, that it is possible to place and keep the patient in the position most favourable to his wound or malady; and secondly, that such an ambulance can be transported up even narrow and tortuous stairs. Another improvement is a two-wheeled ambulance, presented by M. Lagogué, of Alençon, to the Conseil de Salubrité. This is intended to carry two persons; the axle of the carriage being bent at right angles, leaves a large space, from the top to the bottom of the vehicle, between the wheels; and in this space there is room for two "litters" to be suspended by hooks, one above the other. This ambulance can be drawn either by a horse or by one or two men.

A third, the invention of M. Gril, of Poitiers, has also been in use in Paris; and its inventor claims that, even over comparatively rough ground, no more motion is felt than would be occasioned by the gentle swinging of a hammock. It is very light, and can be drawn either by a horse or a man; while the management is stated to be so simple that an entirely inexperienced person would have no difficulty either in lifting a patient on or off.

### A Busy Quarter.

Very familiar have the words, "Three months' course," become to us of late, and apparently the variety of "training" which can be attempted within that limited period is itself practically unlimited. What value can be placed on such "a course" by anyone who has attained to years of discretion it is impossible to divine. However, there must be some demand for cramming in nursing like other work. Otherwise we should hardly hear of a person offering to prepare for the L.O.S., teach dispensing, theoretical and practical district and maternity work, all in three months, for a fee which includes board and lodging during that time.

### The Mechanism of Movement.

### IX.—THE FOOT.

It is very interesting to compare the hand of the ape with that of man, because the two closely resemble each other. In the ape's hand the thumb is much smaller relatively than the fingers, and hence his hand cannot grasp as well as can a man's. If a nut is thrown to an ape he will pick it up, not with the tips of the forefinger and thumb, but between the thumb and the side of the bent fore-finger. In the ape's hand the thumb is very feeble compared with the fingers, which are both much longer and far stronger, and hence the ape uses its fingers much more than its thumb, and when it takes anything it encircles it with its long fingers.

Since trees are the natural homes of the ape, these long fingers are perfectly adapted to encircling the round boughs of a tree. Moreover the whole upper limb or arm of the ape is both longer and more powerful than the lower, to enable it when suspended by its hands to swing itself from one bough to another, or to support its body with its knuckles resting on the ground. The ape uses both limbs equally for progression, and his arm also for grasping or seizing, and hence his upper limbs have more varied uses than those of man.

Let us now consider the extremity of the lower limb, viz., the foot, and see how it is adapted for the purposes which it has to perform, viz., support and progression.

The human foot forms a base or pedestal to the very perfect column made by the lower limb. Moreover, it is so constructed that it can support not only the lower limb, but the weight of the entire body, either in the act of standing or walking. The human foot is as characteristic of man as is the human hand, and its most distinctive feature is the great toe. The first and the last toes are the only two that have any distinguishing names, the great toe is called "hallax," and the "little" toe is specially so-called from its small size.

The great toe is far out of proportion in thickness to the others. It lies parallel to the others, and in its natural shape, when not distorted by ill-fitting boots, forms a continuous line with the inner border of the foot. It cannot be moved like the thumb to form almost a right angle with the hand, and its power of movement is very limited. The great toe is usually the longest, though with some people the second toe is longer.

In its main plan the foot closely resembles the hand, but the foot has only 26 bones as against 27 in the hand; they are arranged similarly, the ankle, corresponding with the wrist, is formed of seven separate bones; beyond are five bones, and the toes consist of 14 bones.

The foot is at right angles to the leg, and so arranged that the sole of the foot is directed towards the ground, and hence we walk on the sole of the foot more or less, but as the sole is arched, not flat, it touches the ground at certain points only, viz., at the heel behind, and at the heads of the bones which form the sole, where they join on to the toes, and more especially that of the great toe in front. At these particular points the skin is much thicker, and is also provided with a pad of fat which acts like a cushion and diminishes the pressure.

With some persons the sole of the foot is flattened, and more of it rests on the ground, but this is not natural, being a deformity known as "flat foot," which exists in almost all people who stand much. It deprives the foot of much of its spring and usefulness, because then the "arch" is lost.

The foot possesses the same properties as the hand, viz., flexibility, elasticity, mobility, and sensitiveness to touch, but the last two in a less degree. The most important property of the foot is its elasticity, which is more necessary to it than to the hand. The elasticity is due to the number of bones and joints which it contains, and also to its arched shape, and is required for two purposes—first, to aid in supporting weight; and, second, to break shock.

When we are standing erect, either on one foot or both, the weight of the body and lower limb is transmitted downwards through the bones of the leg to the upper part of the ankle, where it is divided and distributed in two directions—first, directly downwards through the heel bone to the tip of the heel behind; and, second, downwards and forwards through the bones of the inner border of the foot, to the great toe in front.

The principal arch of the foot runs lengthwise, from heel to great toe, and therefore the weight is chiefly supported by that arch.

The foot is far more liable to concussion than is the hand. Only occasionally is a blow driven at a hard object with the hand, but we constantly strike the hard ground with our feet in walking, running, or leaping, &c. Hence, to duly preserve the body from concussion, those parts of the lower limbs which come into contact with the ground must have an elastic construction such as nature provides them with. In leaping from a height it is very important to remember to alight on the toes instead of on the heels, because if the heels come to the ground first the concussion is at once passed up through the bones of the leg and thigh to the spinal column and head, and, in spite of the buffers in the spine, great shock and injury may be done to the brain. If the toes touch the ground first the concussion is distributed through the joints of the toes, and considerably lessened before reaching the spine or head.

Comparing the foot of the ape with that of man, in the former the great toe is at right angle to the rest of the foot, and hence resembles more closely a thumb. In the ape, too, it is thicker than the other toes, but it is much shorter, and in every way more like a thumb than a great toe.

The foot of the ape is really, as regards its use, a foot-hand, so to say, capable of support, progression, grasping, and holding, though in structure like a foot, consisting of bones and other parts exactly similar to those of a human foot. This power which apes possess of using both hands and feet as grasping instruments has caused some naturalists to call them four-handed.

There is a wide-spread notion that because the right arm is more often used, and on that account is stronger than the left, that the right leg must be stronger than the left, but this is not universally true, though it may be so in some cases. With most people, on the contrary, the left leg is stronger than the right. An eminent philosopher, indeed, attributes man's superiority to the two facts that he possesses hands, but more especially that he has a right hand.

Right-handedness and left-leggedness may be considered as attempts of nature to preserve, as far as possible, a bilateral individual. If everything tended to right-sidedness we should soon become very one-sided objects.

### The Universal Cookery and Food Association.

The trustees and Committee of Management of the above association held a cookery competition at the National Training School for Cookery in Buckingham, Palace Road, on Saturday, July 14th. It was for children varying in age from 10 to 17 years, both boys and girls from the different training schools. The bright and intelligent appearance of the children was most noteworthy, and they seemed to work with considerable zest. Numerous tempting dishes showed the aptitude and skill of the young cooks, and proved that the instruction given, even in the simplest forms of food, ensures that they should be pleasant to the eye as well as palate. Everything was conducted in a most methodical and fair manner, equal chance being given to each competitor. The Hon. Secretary, Mr. Herman Senn, who will probably present the prizes in October, seemed quite indefatigable, and his management of the interesting entertainment was admirable. A sumptuous luncheon was provided for the visitors, and this attention was thoroughly appreciated.

### The Matrons' Council.

ALTHOUGH we have received no official announcement of the date of the meeting at St. Bartholomew's Hospital, at which Miss Isla Stewart, the matron, introduced her proposals, we are enabled, by the courtesy of a correspondent, to publish the following printed statement which was circulated on the occasion. Our correspondents inform us that the proceedings were not unanimous, and that clauses 2 and 3 of the regulations were taken as an indication that the promoters, if successful, may run the Matrons' Council in competition with the Royal British Nurses' Association. This view may be due to the circumstance that the subscriptions of both classes of members are only half the amount now enforced by the Council of the Royal British Nurses' Association. Other correspondents are anxious to know why the new society should be called the Matrons' Council when ward sisters and charge nurses are invited to join it by becoming subscribers of half-a-crown each per annum ?

We defer any comment of our own, but shall be glad to hear the views of our readers upon Miss Isla Stewart's proposals, which were as follows :—

1. That the name of the society shall be "The Matrons' Council."

2. That the objects of the society shall be :
   - (a) To enable hospital matrons to take counsel together upon matters affecting their profession.
   - (b) To bring about a uniform system of education, examination, and certification for nurses in British hospitals.
   - (c) To form an Advisory Committee, to which members can apply for counsel in cases of professional difficulty.
   - (d) To hold meetings to discuss subjects of professional, and also of general, interest.
   - (e) To encourage hospital matrons to understand the methods of procedure at meetings.

3. The society shall be formed of members and associates. Women who are or have been matrons of British hospitals and superintendents of nursing institutions, who are trained nurses, shall be eligible for membership. Night superintendents, ward sisters, or charge nurses of hospitals, who are thoroughly trained, shall be eligible as associates.

4. The annual subscription for members shall be five shillings ; and for associates, two shillings and sixpence.

5. Applications for membership or associateship shall be made upon forms provided for that purpose ; and the election shall be determined by vote at a meeting of the Executive Committee.

6. The honorary officers of the Council shall be a chairman, two vice-chairmen, and an honorary secretary, who shall also act as treasurer. The honorary officers shall be elected at the annual meeting of members in each year.

7. The annual meeting of members shall elect twelve members to form an Executive Committee, which shall carry on the business of the society, and shall organise such meetings and congresses as it may be decided to hold.

8. The Executive Committee shall meet once in each month, or as often as may be necessary, at such times and places as may be decided upon, to elect members and associates, and do such other business as may be necessary.

9. No new bye-law may be made, nor may any standing bye-law be amended or rescinded, except at a general meeting of members, nor unless full notice of such proposed alteration, addition, or omission shall have been given to every member upon the notice convening the said meeting.

### A Sad Accident.

AN inquest was held on the 18th inst. on an infant which met with its death under very sad circumstances at Queen Charlotte's Lying-in Hospital. A dose of carbolic acid appears to have been given in mistake for one of olive oil. The nurse who inadvertently gave the poison took it from a bottle with the label "olive oil" on it, another nurse having filled it in error with the acid. There seems something lacking in the arrangements of a dispensary where confusion of so serious a nature is possible.

### Nursing in Chili.

THE administration of the Chilian Hospital is subject to strange vicissitudes, as one of the first acts of a new Government is to discharge all Members of the Board of Charity, and all hospital doctors who have not upheld the political views of the incoming minister. The disastrous effect of this proceeding, which, according to Dr. Asta-Burrago, is the general rule, can better be imagined than described.

Under a fresh administration doctors, managers and orderlies are promptly discharged and their places given to new comers, to the infinite discomforture of the hospital patients.

Roman Catholic Sisters of Charity are in charge of all the hospitals and have a great deal of influence with the "Administrator." The latter has no payment for his services, he visits the hospital once or twice daily, signs all orders, and sees that order is maintained in all departments. His tour of inspection is, however, always made in the company of the matron or the head nun.

The accounts are kept by the sisters, who purchase the provisions and pay all salaries, supervise all employés, as well as the nursing staff. They also are responsible for the supply of drugs and surgical instruments. "Thus," says Dr. Asta-Burnago, "politics and religion predominate in the management of Chilian hospitals, and science is very little or not at all consulted in their behalf, the consequence of such a state of affairs being that the hospitals in Chili are far below the standard reached by similar charitable institutions in Europe and the United States. The Medical Board is practically a dead body. Its advice is rarely solicited, or not at all, in matters relating to the welfare of the hospital, and any suggestions in that line made by it are generally treated with indifference and even contempt."

No temperature charts are used in Chilian hospitals, and "medical, surgical, gynecological, puerperal, and infectious cases being huddled together in one ward, the doctors practise indiscriminately both branches of general medicine." In some hospitals the resident medical officer "has under his charge six hundred patients during the greater part of the day, and all the night."

The nurse-training school is, of course, non-existent in Chili, and the paid attendants for the patients consist of untrained orderlies and nurses, who are supervised by the religious sisters.

### Novelties for Nurses.

How often does the question of dress overshadow the otherwise pleasant anticipations of a holiday ! "Clothes are such a bother," remarks many a nurse when she discovers that her travelling dress looks shabby and creased as well as out of date after a year's incarceration in a trunk. Under such circumstances a cheap, new, well-fitting gown becomes a necessity for the tired worker, whose spare time and funds seem equally limited, in view of her annual leave. Messrs. J. and A. Philp, Mansfield Road, Nottingham, have set themselves to meet such emergencies as these. They guarantee perfect fit, style, and workmanship in the costumes they provide. Patterns are sent by them on approval with directions for measurement, and if the latter are accurately carried out a customer is supplied with a complete tailor made gown at a guinea or £1 15s. 6d., according to the quality of the material chosen. Nurses will do well to write for samples.

### Under Royal Patronage.

IT has been recently announced that her Royal Highness the Duchess of York has graciously consented to become a vice-patron of Queen Charlotte's Lying-in Hospital, which has already the honour of possessing the Queen for its patron and the Princess of Wales as a vice-patron.

### Notes from Victoria.

(Communicated.)

After all the wrong impressions caused by Professor Morris's injudicious appeal for money from England, and his endeavour to justify his action, it is a treat to hear of hospitals with no debts, and that have no difficulty in raising funds. The Alfred and the Homœopathic in Melbourne are in this case, so are some in country townships. In my last "Notes from Victoria" I mentioned that Daylesford Hospital was to have a new wing for women, for which a legacy of £1,000 had recently been left. Kyneton is enlarging its hospital, with ready money in hand to begin the alterations.

At Castlemaine "they have never been short except once, when a treasurer went off, taking £700 with him." This I learned from Mr. W. Otley, who is dispenser and house steward. He has been in the hospital more than thirty years; has watched its growth, which commenced with a canvas tent, then wooden building, and now a handsome brick and stone building, consisting of two wings, and a block connecting the two. There is also a separate red brick house, erected about three years ago for the nurses. The doctors' house is up the hill a little behind the nurses' house. All looks in excellent order. The matron, Mrs. F. E. Lloyd, was trained at the Alfred Hospital, Melbourne. Some of the nurses are hoping to go there for training and more experience.

There are seven nurses under the matron. One of the doctors gives them lectures three times a week. The nurses are on duty from six in the morning till eight in the evening. One afternoon once a week is free, and every alternate Sunday. Castlemaine is an old mining town. The country is hilly in and around the town, the streets broad, and the air very fine. The Benevolent Asylum is an imposing building of red brick on the top of a hill, with a slope of vineyard in its grounds. There are some trees round the house and an extensive and beautiful view from every window. Twenty-four women have their day and bed rooms up stairs. Two of the youngest and strongest help those who are infirm. One woman is deaf and dumb, but was said by one of her companions to be exceedingly nice and intelligent, "quite superior, you know." The men are more numerous, numbering 82; among these are seven or eight Chinese. They have a large day-room away from the house, where they smoke, read, or talk to their heart's content. Two wardsmen look after the sick and infirm.

### Nursing in U.S.A.

### SHENANGO VALLEY HOSPITAL.

This hospital is situated in Newcastle, Pa., and has been recently rebuilt on the site of the old building, which was destroyed by fire. A corridor connects the operating theatre with the main building, and in a private ward adjoining the theatre patients are sometimes nursed for a few days after operation. The hospital is a small one, containing accommodation for forty patients, and for the nursing staff, and it is beautifully fitted up with the newest conveniences and appliances. The wards contain four or five beds each, the ironwork being painted white, and so are the cots, of which there are three in the children's ward. To every ward a bath-room is attached, and also to the rooms for paying patients. "In front of the surgical ward and women's ward," says the *Trained Nurse*, "are conservatories—one on each side—8 feet wide and 24 feet long. These will be for the accommodation of patients desiring to take a sun bath. The two sun parlours are adorned with growing flowers and plants.

### For Reading to the Sick.

**Motto.**

Alas! by some degree of woe we every bliss must gain;
The heart can ne'er a transport know that never knew a pain.
—*Lyttelton.*

**Verses.**

Amid my list of blessings infinite
Stands this, the foremost, "That my heart has bled";
'Tis Heaven's last effort of goodwill to man;
When pain can't bless, Heaven quits us in despair;
Who fails to grieve, when just occasion calls,
Or grieves too much, deserves not to be blest.—Young.

Lord! dost Thou call this our affliction "light"?
Is all this anguish little in Thy sight?
"Child bring thy balance out. Put in one scale
All thine afflictions; give them in full tale;
All thy bereavements, grievances and fears;
Then add the utmost limit of man's years:
Now put My Cross into the other side,
That which I suffered, when I lived and died."
I cannot, Lord; it is beyond my might:
And lo! my sorrows are gone out of sight!
"Then try another way. Put in the scale
The glory now unseen behind the Veil;
The glory given to be thine own estate;
Use that 'exceeding and eternal weight:'
Which kicks the beam?"
Ah! Lord, Thy word is right;
Thus weighed, my sorrow does indeed seem light.
—*Caroline M. Noel.*

Our pains are portioned to our powers—
His Hand may hurt but cannot harm:
But if the Cross be on us laid, and our soul's crown of thorns be made,
Then, sure, 'twere best to bear the Cross, nor lightly fling the thorns behind,
Lest we grow happy—by the loss of what was noblest in the mind!
—*Lytton.*

**Reading.**

Submission to the will of God, with experience of His support in pain, sickness, affliction, is a more joyous and happy state than any degree of health or worldly prosperity. In pain, sickness, and trouble methinks I hear God saying, "Take this medicine, exactly suited to the case, prepared and weighed by My own hands, and consisting of the choicest drugs which Heaven affords."

Be not disturbed for trifles. By the practise of this rule we should come in time to think most things too trifling to disturb us.

Suffering is an excellent preacher, sent immediately from Heaven to speak aloud in the name of God to the heart, mind, and conscience, and has saved many a soul when, humanly speaking, nothing else could. . . . God is always with me, though I am not with Him; we may learn patience, to lie still and bear. Affliction rightly understood, draws us nearer to our God, stirs us to greater trust in His love: a love that would prove us, bruising only to extract the fragrance. Suffering enhances health. Relieved from pain how grateful and glad we feel. Would that sin were a pain as bitterly felt, as heavy a burden that we might be forced to cry out and plead as earnestly for its relief.—*Ibid.*

Miserable thou art, wheresoever thou be, or whithersoever thou turnest, unless thou turn thyself unto God. . . . For who is he that hath all things to his mind? Neither I nor thou, nor any man upon earth. There is none in this world, without some tribulation or perplexity. Who is then in the best case? even he who is able to suffer something for God. —*Thomas à Kempis.*

And because I do not advert to His presence He sends pain to introduce and even to force me into His company. —*Thomas Adam.*

### Entertainments.

The Late Matabele Campaign.—Most interesting was the lecture given by General Digby Willoughby at St. James's Hall on July 10th on "The Matabele War," in aid of the Memorial Fund which has been formed with the object of building a hospital at Buluwayo to the memory of the brave men who fell and died in the course of the campaign. Considerable excitement was caused, on the introduction of the lecturer by the Duke of Abercorn as a most distinguished officer, by an individual in academic garb, who loudly demanded from the body of the hall to know if General Willoughby held a commission in the British Army. Receiving no reply, he continued to shout somewhat incoherent accusations against the Chartered Company, excitedly brandishing the while a stout staff which he carried, and he was at last forcibly removed struggling violently. At the conclusion of the lecture he might have been seen standing in the entrance hall distributing a pamphlet of his own writing, "Sham Science," which, we may add, was chiefly remarkable for its extraordinary confusion of phraseology, and which revealed his name to be Mr. G. P. Mansel Weale. General Willoughby in his lecture gave a short history of Matabeleland and Mashonaland, the people, and their habits and customs. He gave sketches of the lives of the chief officers who took part in the war, most of which were illustrated by limelight portraits, those of Majors Forbes and Wilson, of Mr. Selous and Mr. Rhodes, evoking immense enthusiasm amongst the audience. Other illustrations were also given, notably two pictures painted by Mr. C. Woodville for the benefit of the fund. An account of Dr. Jameson, the present Administrator, who has done so much in the service of the Chartered Company, was also greeted with great applause. The story of the tragic fall of Major Wilson and his little band of brave men, as graphically told by General Willoughby, must have stirred the hearts of all present, and if we may judge by the enthusiastic interest manifested by the audience the other night, the funds collected should be large. No better memorial to the heroes of the Matabele War could there surely be than the proposed hospital, and we cordially and earnestly wish the project every success.

The French Hospital.—The charity reception held on Tuesday night at the Grafton Galleries in aid of this hospital may be fairly ranked among the leading events of the season. The committee are to be congratulated on an almost unprecedented success and on the boldness of their project. By eleven p.m. the galleries were fairly thronged, but it was not until a considerably later hour that the entertainment commenced, when the programme opened with a recitation by Mr. Irving. This was followed by two shorter ones by Miss Ellen Terry, who looked as youthful and blooming as the roses in her hair. Madame Albani sang twice, generously responding to an encore, the long music room lending itself well to her full rich voice. There were one or two other items in the programme, and then Madame Sarah Bernhardt and Madame Réjean appeared. The latter was quite inimitable in her rendering of "La Pupée," and adequately sustained her character of the greatest comedienne of the day—versatile, piquant, enchanting—and she held her English audience as in Paris she holds the Parisians. Then the great Sarah came on the daïs, whose magnetic presence had already made itself felt among the audience. In "La Pluie et le beau Temps," which she played with Monsieur Guitry, Madame Bernhardt was inspired and quite above all criticism. In a word she was at her best and looking her best, resplendent in most gorgeous attire. Apart from the unprecedented attractiveness of the programme the entertainment proved a financial success and a substantial benefit to the hospital funds, which were much in need of such generous help. Amongst the brilliant assembly we noted members of the French Embassy, the Italian Ambassador, Sir William MacCormac, Sir W. Rose, the Hon. Mrs. Lancelot Lowther, Lady Colin Campbell, and Madame Melba. The donations included twenty guineas from Mr. Irving and five guineas from Miss Terry. Dr. de Vintras, the able secretary of the hospital, is to be much congratulated on the excellent arrangements and the success of one of the most brilliant entertainments of the season.

### The Workhouse Infirmary at Bishop Auckland.

In pleasing contrast to the recent painful disclosures regarding neglected and harshly-treated paupers, comes a report of the kindly tone prevalent at Bishop Auckland Infirmary. The matron has imparted a similitude of "home" to the "House," which is seldom to be found in such institutions, however excellent the administration may sometimes be.

The Commissioner of the *British Medical Journal* commends the cooking, the beds, the kindness, and the cleanliness, but skilled nursing is conspicuous by its absence. There is one trained nurse, but as she is the midwife, besides having the charge of some seventy patients, her personal attention to each is obviously out of the question.

There is no night nursing whatever, and when there are any infectious cases they are placed in the isolation ward, under the sole charge of a pauper attendant.

An additional day nurse and a permanent trained night nurse are suggested by the Commissioner as quite necessary to secure for the patients anything like adequate attention. There are urgent cases of illness as well as operations to be considered, the infirmary furnishing the only form of hospital accommodation in Bishop Auckland. It is therefore obvious that pauper assistance is utterly inadequate to cope successfully with the nursing branch of the workhouse.

The board are certainly to be congratulated on the master and matron they have secured, for their affectionate care of the inmates leaves little to be desired, and it is pleasant indeed to read of cushioned armchairs for the poor old people, and of warm shawls, slippers, and dressing-gowns for invalids. When the important and essential addition of an efficient staff of trained nurses is made, the sick paupers at Bishop Auckland will have every reason to be grateful for the provision made there for their comfort.

### A Case for Assistance.

This case is one which, after thorough investigation, has proved most deserving of help, and we hope that Miss Bell and Miss Farrow, of the Home for Aged Mariners, Egremont, Cheshire, will receive many subscriptions to the fund. They acknowledge with thanks 5s. from Miss Lucy White, of Constantinople, this week. The nurse whose cause these ladies have so kindly espoused was trained at King's College Hospital, and for many years filled the post of matron in London and at provincial hospitals. Misfortune and ill-health have reduced her to poverty, and she is now endeavouring to free herself from debt and to stock a small shop at Cardiff, where she might earn her own living. All particulars can be learnt from Miss Bell or Miss Farrow.

### Notes and Queries.

### Queries.

(100) *Dispensing.*—Kindly tell me how to get coaching in this.—*Dispenser.*

(101) *English Abroad.*—I should be very glad if you could give me some information about English hospitals abroad.—*Marjorie.*

### Answers.

(100) *Dispensing* (*Dispenser*).—Read chapter with this heading in Burdett's Hospital Annual, Scientific Press, 428, Strand. Price 5s.

(101) *English Abroad* (*Marjorie*).—Your question is a very vague one. Burdett's Annual will give you much information; also two letters in "Everybody's Opinion" this week.

### Nurses' Holidays—Southend.

When a short time is all that can be spared for a holiday, the point most desirable to consider is how small a portion of it need be devoted to the railway journey.

Southend seems to meet our wishes in this particular; it is a pretty little place at the mouth of the Thames; it is reached from London in about an hour, and the fare is insignificant (just over two shillings), and it enjoys the reputation of being a cheap place. But alas! for this reputation, lodgings are quite as expensive as at more fashionable places, two to three guineas being asked for two bed-rooms and a sitting-room, and thirty shillings a week for one bed-room and a sitting-room.

These appear to be the usual prices, but in August there seems to be no limit to which they may not reach, should the season be a good one. The houses are small, and not many of the dozen or twenty inspected came up to a London nurse's idea of cleanliness. A bath-room is an almost unknown luxury.

There appear to be some comfortable boarding houses where large, clean double rooms can be had for two and a half guineas a week (and this includes liberal board) and single rooms for thirty shillings. Clearly, therefore, for one visitor, or for two sharing a room, a boarding house would be cheaper than lodgings, and the occupant would be spared the daily anxiety of ordering "what you please," as the landlady smilingly remarks.

Provisions are about the same price as in London, though fruit is very dear and inferior. There is an excellent supply of fish, some of it local and some coming daily from Billingsgate. The water is deliciously soft, as the state of our hands very soon testified, and the washing of clothes is excellently done, the linen returned by the laundress being of an almost original whiteness—a consideration when to look fresh and clean, as well as to be it, is one's desire. But this same soft water makes execrable tea; instead of our accustomed refreshing drink, we get a very black decoction, tasting strongly of carbonate of soda, and with no trace of tea about it. Having personally prepared it, we knew it should be all right, and so we consult the local chemist, but all the explanation that he could offer was that the water was unusually soft, and for a remedy he suggested putting in half the quantity of tea. But this remedy had no satisfactory results. The water is drawn from deep wells, and is considered almost pure; most of its salts in solution are not only wholesome but beneficial to visitors with "livers."

The place is laid out somewhat after the manner of Folkestone, there being a charmingly breezy walk well supplied with seats all along the top of the cliff, which extends for some distance.

The boats are a decided feature; all shapes and sizes are anchored near the shore, ready to take visitors out by the hour, and there are also some pretty cutters which, at sixpence a head, take about twenty people for a sail across the estuary to Sheerness or Queenborough; of the ordinary sixpenny steamer, with its surging crowd of noisy passengers, the place appears guiltless. In its place, the pier seems to be occupied by the majority of the day trippers; it is a mile and a quarter long and has an electric tramway running its whole length. One hears people stoutly declaring that "they would not dream of riding that little distance," and they set off to walk with a virtuous look upon their faces; before long, however, their limbs begin to drag wearily, and the virtuous look fades as they pace along the black planks, which seem to stretch on into eternity. More than one weary pedestrian wishes that the cars had a station half way, and another has a lurking hope that it may be possible to hail them like the ordinary tram, but alas! for their hopes, the cars rush unheeding by them, and all they can do is to assure each other, in a tone which shows they do not in the least believe it themselves, that the end must come soon, and on they patiently trudge. When the end is reached at last, the breeze is pleasant and invigorating, and the water splashing and gently rippling round is of a proper sea-green colour. Away in the distance the sea can be seen, and the big ocean-going steamers appear quite close as they pass in front of us. There is an immense amount of interest to be got out of these passing vessels, with their distinctively painted funnels. At night especially they look like huge black one-eyed monsters relentlessly and silently going on their way, and carrying their victims to unknown destinies.

The walks round Southend are unusually pretty. Canuden, about six miles off, is well worth a visit; it has a curious old church, the tower of which was used in former times as a beacon, it is supported by such massive buttresses that its appearance is more that of a castle keep than of a church tower.

In another direction, for a twopenny train fare, we can get to Rochford, the former home of the Boleyns, where the beautiful Ann, Henry VIII.'s second queen, was born. Much of the house has been pulled down, but the remaining rooms with their old oak panelling are full of interest, and are shown to strangers.

Coaches with "garden seats" at a shilling each take visitors drives in all directions; by this means a good idea is obtained of the neighbourhood, and we get a glimpse of many a quaint old-fashioned village such as we read of, but do not often see in these days. E. G.

### Where to Go.

Twelve Days' Swiss Holiday via Dover, Calais, and Paris.—The sum of £10 10s. includes a second-class return ticket to Grindelwald, meals on the outward journey, a week's accommodation at Grindelwald, Scheidegg Pass, Meringen, Grimsel Pass, Rhone Glacier, Goeschenen, and the St. Gothard. Economical extensions arranged to Mont Blanc, the Matterhorn, the Italian Lakes and Falls of the Rhine. These parties are specially arranged to secure economy in travelling; the right to return at any time within 45 days, and to break the journey at any of the main points of interest. Parties start every Tuesday and Friday during the summer. The following dates are open for bookings: July 17th, 20th, 24th and 26th; August 3rd, 8th, 9th, 10th, 14th, 17th, 21st, 24th, 28th and 31st; September 4th, 7th, 11th, and 14th. Booking fee, one guinea. All further particulars can be obtained from Mr. T. H. Bishop, Secretary, 5, Endsleigh Gardens, London.

The Grindelwald Conference, under the presidency of the Rev. Dr. Henry S. Lunn, in Section III., to be held July 29th to August 10th, will deal with the Union and Church Problem. Papers and sermons by the Deans of Armagh, Bristol, and Norwich, Prebendaries Grier and Webb-Peploe, Canon Hammond, Professors Shuttleworth and Lindsay, Dr. J. Monro Gibson, Dr. Glover, and others. Section IV., from August 11th to September 7th, educational, scientific, and literary lectures will be given by Sir Benjamin W. Richardson, Sir Robert Ball, the Rev. Hugh Price Hughes, the Rev. Charles Berry, the Rev. W. J. Dawson, Messrs. Edmund Gosse, Harry How, Carus Wilson, Edward Whymper, Miss Friedrichs, and Mrs. Fenwick Miller. Concerts are also arranged in which Mrs. Mary Davies and others have promised to assist. Section V., which will last from September 9th to 17th will be devoted to matters historical and political.

### Wants and Workers.

Can any readers help a poor mother to get her boy into the Home for Crippled Boys, Kensington, or, failing that, to obtain "irons" through the means of a letter for the Surgical Aid Society? Address Cripple, care of the Editor of The Hospital, 428, Strand, London, W.C. [Has our correspondent tried to obtain the irons for this case through the intervention of the clergy of the place of worship the poor mother attends? If not, she should be advised to apply to one of them for a letter to Mr. H. N. Custance, Secretary of the Metropolitan Hospital Sunday Fund, Mansion House, E.C.—Ed. The Hospital.]

☞ For The Muses' Looking Glass, Everybody's Opinion, Book World for Women and Nurses, &c., see page clxix. et seq.

### The Muses' Looking Glass.

### ST. GEORGE'S HOSPITAL.

One is inclined to wonder if nurses at St. George's, in bygone days, were ever tempted to break rules in order to take part in the delights of May Fair.

"I wish you had been at May Fair," wrote one Brian Fairfax from Lockett's to a friend in Yorkshire, in the year of grace 1701, "when the rope dancing would have recompensed your labour. All the nobility in the town were there. There was the city of Amsterdam, well worth your seeing; every individual house was carved in wood, in exact proportion one to another; the Statdhouse was as big as your hand."

The *Tatler*, No. 20, informs us that though May Fair was dwindling "it is still allowed to sell animals there, therefore if any lady or gentleman have occasion for a tame elephant, let them inquire of Mr. Pinkethman, who has one to dispose of at a reasonable rate. The downfall of May Fair hath quite sunk the price of this noble animal."

May Fair was instituted in honour of St. James; it opened in the month of May, but lasted six weeks, and it was held where Curzon Street and Hertford Street now are.

In the year 1708 a Mr. Sheppard received "for the ground rent of the faire, market, and one house, £1 1s."; but stimulated by the virtuous example of the City with regard to Bartholomew Fair, in this same year the Grand Jury of Westminster earnestly desired the termination of the "public nuisance . . . the yearly riotous and tumultous assembly in a place . . . called May Fair."

The death of a certain Hugh Audley gentleman, of the Inner Temple, in 1662, was the occasion of the publication of a pamphlet entitled "The Way to be Rich, according to the practice of great Audley, who began with £200 in the year 1605, and died worth £400,000, this instant, November, 1662."

In days when the Penal Laws were still in force against Roman Catholics, the Portuguese Embassy had a chapel in South Audley Street, and here, according to the Catholic rite, David Garrick, the actor, was married to Eva Maria Violette on June 22nd, 1749.

Gibbon the dutiful, "who sighed as a lover and obeyed as a son," had given his father cause for anxiety before his admiration for the Swiss lady, whom he allowed to become Madame Neckar. In his Oxford days he had been received into the Church of Rome, and to counteract this influence Mr. Gibbon, sen., sent him to live with Mallet, the poet, whose atheistical lessons were easily imbibed by the future historian. Mallet lies buried in the cemetery of Grosvenor Chapel, with Ambrose Philips, another poet, John Wilkes, the "Friend to Liberty," and all that remains of lively Lady Mary Wortley Montague.

Lady Mary always had a dread of growing old, and old age attacked her in an ugly way. Horace Walpole says, "She wears a foul mob that does not cover her greasy, black locks, that hang loose, never combed or curled; an old blue . . . wrapper, that gapes open and discovers a canvas petticoat. Her face . . . partly covered . . . with white paint, which for cheapness she has bought so coarse that you would not use it to wash a chimney." "Such is one of the latest portraits of the woman who had been a poet's idol and the cherished beauty of a Court."

Grosvenor Square was the home of another famous lady whose ugliness was her birthright, and not the result of decay, George I.'s mistress, the Duchess of Kendal. She had a daughter, who became the wife of the witty Lord Chesterfield, and they also lived in Grosvenor Square.

This was the Earl at whose doors the great dictionary maker was forced to cool his heels, and upon whom he took such revenge. Did he, on visiting in after years his friend Mrs. Thrale in this square, call to mind the humiliations he had endured in the great man's ante-chamber?

In Grosvenor Street lived Mrs. Oldfield, and "Butcher Cumberland," of Culloden fame, died there; but to mention all the names which suggest histories would be impossible; therefore, turning our attention to Piccadilly, we may recall the curious wedding of James, the fourth Duke of Hamilton, to the youngest of the famous and beautiful Miss Gunnings. Walpole says the ceremony took place in Keith Chapel, May Fair, half an hour after midnight, and was performed with the aid of a bed-curtain ring.

The earliest known reference to Piccadilly is botanical. In 1596 Gerrard, in his "Herbal," states "that the small wild buglosse grows upon the drie ditch banks about Pickadella."

At the north-east corner of the Haymarket in the seventeenth century there was a large house called Piccadilly Hall. It was inhabited by a charitable personage named Robert Baker, who seems to have rather resented the name given to his house, on account of its reference to a tailor, who made the necessary money to build it by the sale of piccadellies, a kind of stiff collar. Much has been written about the etymology of the word Piccadilly; *picca* is the Italian and Spanish for a spear head, and it has been suggested that the stiffened plaits of the piccadel possessed a fancied resemblance to the bristled points of these weapons.

In Clarendon's "History of the Rebellion" Piccadilly Hall is described as "a fair house for entertainment and gaming, with handsome gravel walks with shade, and where are upper and lower bowling-greens, whither many of the nobility and gentry of the best quality resorted, both for exercise and conversation." In Panton Street we have preserved the memory of the sale of Piccadilly Hall by Robert Backer's widow to Colonel Panton.

Evelyn records in his "Diary" for July 31st, 1662, that he "Sat with the commissioners about reforming buildings and streets of London, and we ordered the paving of the way down St. James's North, which was a quagmire, and also of the Haymarket about Piquidillo."

In 1757 Piccadilly between Devonshire House and Hyde Park Corner resembled Euston Road of to-day, being given up to stoneyards of statuaries.

"And now from Hyde Park Corner come
The gods of Athens and of Rome;
Here equally Cupids take their places
With Venus and the clumsy Graces."

Two years later Walpole wrote, "I stared to-day at Piccadilly like a country squire; there are twenty new stone houses."

The husband of Nelson's Lady Hamilton died at No. 99 Piccadilly, and the White Horse Cellar, the starting-place for coaches, was named in honour of the accession of the House of Hanover.

This suggests to us that St. James's Street, close by, was the centre of eighteenth century political life. The last house but one on the south-west was the St. James's Coffee House, the rendezvous of the Whigs. In Queen Anne's reign a waiter, "one Mr. Kidney," "long conversed with and filled tea for the most consummate politicians" here.

From here also the *Tatler* dated, and it was at the St. James's Coffee House that Swift found Stella's letters waiting his leisure and inclination to fetch them.

The Tory Cocoa Tree was also in St. James's Street, and the owner of Ozinda's Chocolate House, next to the Palace, was arrested in 1715 "for supposed complicity in Jacobite conspiracy."

Just a kindly thought and smile for Garrick's "Odd Fellow," the "scholar, rake, Christian, dupe, gamester, and poet," who wrote the "Retaliation" on account of a dinner in St. James's Coffee House, and we must wake up to the nineteenth century and the fact that the two hours off duty are over.

### Everybody's Opinion.

[Correspondence on all subjects is invited, but we cannot in any way be responsible for the opinions expressed by our correspondents. No communications can be entertained if the name and address of the correspondent is not given, or unless one side of the paper only be written on.]

### THE "NURSING RECORD" AND ITS "POETRY."

"F.R.C.S." writes: I observe that the *Nursing Record* is in a curiously uncertain condition of mind; not knowing whether its chief feeling should be anger at my exposure of its literary shortcomings, or surprise that I should take the trouble to examine its pages. In this difficulty it certainly breaks "gentle concord"; and I greatly fear that, "when time unveils eternity," it will not be in a position to "plead," even "silently," for its presumptuous critic.

I will endeavour to remove all occasion for surprise. I look at the *Nursing Record* because I desire to keep myself informed about the work and prospects of the Royal British Nurses' Association, of which, in the long intervals between the appearance of its own journal, the *Record* seems to be the appointed organ. I find in the *Record*, week after week, what I do not find anywhere else, namely, an official statement, signed by the secretary to the association, a sort of irregular "Court Circular," telling the world what the association has been doing or intends to do; and I return to the subject of this letter in order to express my doubt whether the rulers of the association are well advised in permitting this official statement to be continued, at least if it is to be confined to the *Record*. I dwelt only on the comic side of the ludicrous blunders in the "poem"; but it should not be forgotten that there is a serious side to them as well. The lines which the *Record* paraphrased were written to convey the strongest possible assertion of a particular theological view, namely, that eternal happiness is to be secured by good works alone, irrespective of faith or doctrine. This view, which is held by many good people, is as strenuously opposed by others; and, as nurses may be of all shades of religious opinion, it seems hardly prudent for a nursing journal to take any side in such a controversy. The writer of the original verses, however, made Christianity an implied condition of the benefits which the good works were to secure; but the *Record* has struck out this implied condition, and, by substituting "social" for "Christian," has placed itself in opposition to the whole body of Christian doctrine, wherever or by whomsoever held. I do not know enough of the "religion of humanity" to be able to say whether the word "social" in this connection amounts to a profession of Positivism, but it may certainly be understood as a repudiation of Christianity; and I should not be surprised to find that many good nurses, who are also good and pious women, are greatly shocked at the partnership in which the Royal British Nurses' Association seems to have engaged. At all events, the propriety of continuing this partnership seems to be a matter which should occupy the attention of those who are most concerned.

Personally I am glad to be able to confirm the surmise of the *Record* that I am blessed with sufficient leisure to be able now and then to take account of its proceedings. I profess no acquaintance with "Satan," and cannot speak of his opinions with any certainty, but, judging from common report, I do not think he would rejoice at the unmasking of an impudent imposture, or would regard such an action as being at all the kind of "mischief" proper to be found for "idle hands." But the *Record* probably understands this part of the question, and I will not dispute about matters which are too high for me.

### MADEIRA.

"Enquirer" writes: I hear there are nursing institutes at Madeira which are much recommended. Could any reader of The Hospital kindly give me particulars with regard to the same, also the addresses?

### HOLIDAY HOMES.

"Teddington" writes: I and another nurse have just returned from a visit to "District Nurse," who recently in The Hospital invited nurses needing a rest to visit her in Cheshire. "Nurse Mary" is a charming hostess, a lady; and in her cosy home any lady might make herself happy. I cannot speak too highly of the pleasure and benefit we both derived from our stay in the country with her. The return fare from Euston is 29s., and the only terms are 5s. to 7s. per week. All particulars can be obtained from Nurse Mary, 12, Teddington Park Road.

### NURSING ABROAD.

Miss Pearson, of Les Agaves, Cannes, writes from Châlet Anca, Aulliens sur Aigu, Suisse: In answer to Nurse B's inquiry, I should like her to know that I have for many years had out nurses every winter to work among the English invalids. Some of these live in my house, others are lodged with my friend Mrs. Dan Norris (formerly head of St. Mary's, Paddington). Besides our nurses, the Hollond House at Nice has a branch establishment at Cannes, and the Society of the Holy Cross (R.C.) has also a home for nurses. If Nurse B wishes for further information, she can apply either to me or to Mrs. Norris. This address will find us till end of September.

"A Visitor to San Remo" writes: Seeing questions in your columns from nurses wishing to go abroad for the winter, I am writing now to tell them of a nursing home at San Remo where seven nurses were employed last winter. I think your correspondent, supposing always that she is a thoroughly efficient as well as a qualified trained nurse, would do well to write for particulars to the Superintendent, now in England, Miss Briant-Hurst, Maidstone.

### TRAINING.

"Matron" writes: I am anxious to find out if it is possible for a girl to get training either in a London or country hospital who has had the first joint of index finger amputated she being most suitable in every other respect to train as a nurse. Would a cottage hospital take her?

### Enthusiasm at Dartford.

A most attractive bazaar at Dartford was opened the other day by the Countess Stanhope, accompanied by Earl Stanhope, Lord Lieutenant of the county. Its object was the raising of funds for the cottage hospital which is now nearing completion, and £1,000 subscribed by Mr. Silas Burroughs has been supplemented with other sums to a similar amount, only £1,500 being still needed. The weather was fine and the attendance of visitors excellent, and the speakers referred with great satisfaction to the liberal and enthusiastic support which the new hospital was receiving from the working men of the districts. The need for such an institution has long been realised, and there is every prospect that the work so auspiciously commenced will arrive at a triumphant conclusion.

### Appointments.

Royal Derby and Derbyshire Nursing and Sanitary Institute.—Miss Agnes Atthill has been appointed Lady Superintendent at this Institute. She was trained at St. Mary's Hospital, Paddington, and we wish her success in her new work.

Solihull Union.—Miss Pleasant Allen, who was Head Nurse of the male infirmary of the Minster Workhouse, has been appointed Matron of the Solihull Union. Miss Allen has been for sixteen years in the service of the Minster Guardians, and they speak in the highest terms of the faithful work done by her. She takes many good wishes to her new post.

### The Book World for Women and Nurses.

[We invite Correspondence, Criticism, Enquiries, and Notes on Books likely to interest Women and Nurses. Address, Editor, THE HOSPITAL (Nurses' Book World), 428, Strand, W.C.]

THE STORY OF DAN. 1 Vol. By M. E. FRANCIS. (London: Osgood, McIlvaine, and Co. 1894.)

The scene of this simple and charming story is laid in Ireland, and the characters are some village people, a priest, and a farmer and his sister. The local colouring is delightful. There is no word of moonlighting and political agitations, those tiresome items of the stock-in-trade of the common Irish story writer. It is doing the author the highest justice to say that her plot might have been worked out in any country, among any set of people, for the story deals with one of the saddest and yet most attractive possibilities of common human nature—the possibility of self-sacrifice for a worthless object. In this case the object is a beautiful but slatternly Irish girl, whose sole redeeming feature is her devotion to an idiot brother. A simple peasant is in love with her, and you must read the story to see how she accepts him casually because there was no special reason why not. How she became dazzled by the hope of marrying a small farmer, and accepting every sacrifice on the part of her lover, laughed at the misfortunes she had led him into. The story ends with a crowning piece of self-abnegation on his part, and he leaves the world in disgrace, actually killed by the horror of a false accusation of murder which he had brought on himself to spare the feelings of his faithless love. The good priest, who is the friend and guide of his flock, is not the least well-drawn character in the book. We have told as little as possible of the plot of this book because we do not want to spoil the pleasure of those who will read it. Simply as a story it is most absorbing and very cleverly worked out. But it is more than this, for although there is no attempt to convey a moral, everyone will be the better for reading a book in which the higher possibilities of human nature are portrayed.

RAMBLES IN BOOKS. By CHARLES F. BLACKBURN. (London: Sampson Low, Marston, and Co. 1894.)

We must confess we fail to see the precise object which the author of this singularly unnecessary book hoped to attain by its publication. It consists of a catalogue of the books comprising the writer's library, with scrappy extracts therefrom, and comments and remarks of his own attached, which are neither instructive nor amusing. Such a note, for instance, as the one appended to "The Wreck of the Grosvenor"—"when this first appeared I copied out whole passages in sheer admiration"—which we take at random as a fair example of the style of comment throughout the book, can be of no interest to the general reader, or indeed to any one, except the writer or his immediate friends, and Mr. Blackburn would have been better advised, we think, had he refained from thus swelling the already appalling number of publications which are daily issuing through the press. The best part of the book is its binding.

### MAGAZINES FOR THE MONTH.

### THE NEW REVIEW.

The "New Review" for July is a first rate number. *Apropos* of M. Sardou's play, Mr. Vandam provides an article on "The Real Madame Sans-Gêne," who it appears was not the Maréchal Lefebvre at all but Therèse Sutter, née Figueur, who figured as a trooper in the wars of the Consulate and Empire under the name of Sans-Gêne. She performed valiant service, to which her sex, which was well known to her comrades, does not appear to have been any bar, became, through the good offices of Napoleon, whom in his earlier days she had roundly abused for a "blackamoor," the secretary and reader of Madame Augereau; and before her death in 1861 gave her memoirs to the world. Following the fashion of criticising the critic, Mr. Bernard Shaw, in the same Review, holds forth in defence of "Arms and the Man"—somewhat of an act of supererogation, surely, since a good play, like good wine, speaks for itself; and Mr. Dudley Hardy, Mr. Aubrey Beardsley, and M. Jules Chérêt expound their several views on "The Art of the Hoarding.'

### THE NINETEENTH CENTURY.

Mr. Swinburne's lines on the death of President Carnot occupy the place of honour in the "Nineteenth Century;" and another recent death, that of the Sultan of Morocco, has inspired Lord Meath to write of the "Land of Incredible Barbarity." The title of the article is justified by its contents, which as a revelation of extortion and cruelty it would be hard to beat. Mr. T. L. Bullock writes on "Competitive Examinations in China," drawing a curious parallel between the education of the Chinese scholar—which consists in the study of ancient literature in what is, to all intents and purposes, a dead language—and the classical education of the European.

### THE FORTNIGHTLY REVIEW.

M. Paul Verlaine's "Notes on England," in the "Fortnightly Review," are a kindly appreciative account of his experiences as a tutor in an English school. Especially good is the little French and English conversation which took place at his first meeting with the principal of the school; and one is glad to hear that he found the English Sunday "not so terrible after all." The Rev. H. R. Haweis' contribution on "The King, the Pope, and Crispi" is a summary and forecast of the much involved present and future of Italy; and Mr. Oscar Wilde sends some allegorical "Poems in Prose," which in their own way are gems of thought and imagery.

THE YELLOW BOOK has made its second appearance in our midst, and volume two of this much-esteemed quarterly is placed before us for review. Without in any sense wishing to detract from the merits of its predecessor, all will agree that a distinct improvement, both in its literary and artistic section, is observable in the present quarter's production. Whilst perhaps in the letterpress there is no one specimen of what one might call the "supreme" in literature, yet there is a very excellent standard maintained throughout: everything in this July issue is well worth reading. Firstly, there is some good fiction—short stories from the pens of Henry Harland, Hubert Crackenthorpe, Henry James, Ella D'Arcy, and others, and if there is too little of more serious and graver matters, the fault lies in the quantity not the quality. Among this latter of the non-fictional order we find "The Roman Road," by Kenneth Grahame, the most attractive; and yet the question arises in one's mind as one reads, Is this not fiction, too; and that one has been misled after all by its suggestiveness and sense of reality into placing it in the realms of a sterner reality? But whether as romance or reality, Mr. Kenneth Grahame shows us in "The Roman Road" a fascinating study. "Madame Réjane" has a rhapsody all to herself at the hands of Dauphin Meunier where "a fabulous being in everyday human form" is shown to us, certainly in the most flattering of pen portraiture. First and foremost among the drawings in the "Yellow Book" comes what Mr. P. Wilson Steer calls "a portrait of himself." In the background there is a three-quarter figure of a man at an easel, a man with no head nor features. In the immediate foreground sits a young girl in negligé attire, a shock of curly hair tumbling over her face and shoulders; the attitude, the drawing is inimitable, but one makes the matter-of-fact inquiry, "where does the portrait come in?" As a picture the grouping, the treatment is quite admirable, but as portraiture Mr. Wilson Steer has certainly extended the more usual and definite sphere of that particular art. We have always considered this artist a rising—and more recently a "risen" man, but this "portrait" of himself—the representation of a headless man and a fair young model—fairly puzzle one. Mr. Aubrey Beardsley has six pen drawings, Mr. Serjeant a profile drawing of Henry James, and Mr. Walter Sickert some very clever studies.

